# Automodified Poly(ADP-Ribose) Polymerase Analysisto Monitor DNA Damagein Peripheral Lymphocytes of Floriculturists Occupationally Exposed to Pesticides

**DOI:** 10.3390/cells8020137

**Published:** 2019-02-08

**Authors:** Serena Imperato, Carmela Mistretta, Maria Marone, Ilaria Migliaccio, Ilaria Pulcinelli, Maria Rosaria Faraone Mennella

**Affiliations:** Department of Biology, University of Naples “Federico II”, Via Cintia, 80126 Naples, Italy; serenaimperato@gmail.com (S.I.); carmela.mistretta@libero.it (C.M.); mariamarone1990@gmail.com (M.M.); ila.migliaccio@studenti.unina.it (I.M.); il.pulcinelli@studenti.unina.it (I.P.)

**Keywords:** Poly(ADP-Ribose)Polymerase, automodified PARP (PAR-PARP), DNA damage, pesticides, growers, greenhouses

## Abstract

Increased DNA damage and the propension to cancer development, depend on the modulation of the mechanisms to control and maintain genomic integrity. Poly(ADP-Ribose)Polymerase activation and automodification are early responses to genotoxic stress. Upon binding to DNA strand breaks, the enzyme, a molecular DNA nick sensor, is hyperactivated: this is the first step in a series of events leading to either DNA repair or apoptosis. Enzyme hyperactivation and automodification can be easily measured and are widely used to look at DNA damage extent in the cell. We investigated whether these two markers (increased catalytic activity and auto modification), could help to monitor DNA damage in lymphocytes of flower growers from Southern Italy, occupationally exposed to pesticides. Peripheral lymphocyte lysates were analyzed for Poly(ADP-Ribose)Polymerase activity, and by SDS-PAGE and anti-Poly(ADP-Ribose)Polymerase 1-antibodyto measure automodified Poly(ADP-Ribose)Polymerase levels bydensitometry. Poly(ADP-Ribose)Polymerase activity and PARP automodification followed the same trend. Growers daily exposed to pesticides, showed both biomarkers very high, either in the presence or in the absence of pathologies. PARP activity and auto-modification in peripheral blood lymphocytes are possible, non-invasive, androutinartools to monitor the healthy conditions of floricoltorists.

## 1. Introduction

DNA structural alterations play a primary role in several pathologies, including neoplastic transformation [1,2]. Many experimental evidences indicate that increased DNA damage, and then propension to cancer development, is related to genetic factors as the modulation of the complex mechanisms involved in the control and maintenance of genomic integrity [3]. PARP-1 activation is an early response to genotoxic stress [4]. Infact, the enzyme acts as a molecular DNA nick sensor binding DNA strand breaks with high affinity [5]. This interaction stimulates PARP-1 catalytic activity and is the first step in a series of events leading to either DNA repair or apoptosis (reviewed in [5,6,7]).

On such a background, it is clear that DNA damage, as well as alterations of some parameters of the poly-ADP-ribosylation system (PARP hyperactivation and automodification) can be considered as valuable biomarkers to assess the risk of cancer development in individuals exposed to genotoxic agents. In fact, a general and central task for worker protection is biomonitoring of occupationally exposed people [8,9]. For agriculturist category, hindrance to perform a right control of risk levels are several variables, i.e., mixing agrochemicals, loading equipment, spraying and application of insecticides, absorption from dermal exposure or inhalation, improper use of pesticides, use of mixtures of different compounds, etc. [10]. So many factors do not allow to confirm the associations between the chemical substance and the disease suggested by epidemiological studies. Acute toxic effects are easily recognized, whereas the effects resulting from long-term exposure to low doses are often difficult to distinguish. However, a growing evidence has confirmed that chronic exposure to pesticides may involve alterations of various organs and systems of the human organism such as the nervous, endocrine, immune, reproductive, renal, cardiovascular, and respiratory ones; therefore, an increase in risk is documented for multiple pathologies such as: cancer, diabetes, respiratory diseases, neurodegenerative diseases, cardiovascular diseases, disorders of the reproductive sphere, metabolic and hormonal dysfunction (thyroid), with different molecular implications [11,12]. Attention is payed to consequences for human health from ‘chronic’exposure to pesticides, i.e., exposure to small and prolonged doses over time whichdoes not concern only the population exposed toworking reasons, but it now concerns the whole general population [13,14]. The etiopathogenetic mechanisms of the related pathologies are free radicals, endoplasmic reticulum stress, endocrine interference, neurotoxicity, etc., although of particular importance remains the action of carcinogenicity [14].

Genotoxic effects are considered among the most serious of the possible side effects of agricultural chemicals [13,14,15,16,17]. However, the absence of a systemic surveillance of agriculturist health does not allow an actual estimation of exposure levels and pesticide-dependent diseases. Here because it is essential to have reliable and relevant noninvasive biomarkers to improve biomonitoring, and treatment of diseases caused by, or associated with, genetic damage. Evaluation of DNA damage in agricultural workers exposed to pesticides has been performed by different methods (polymorphism analysis, comet assay, chromosomal aberration, sister-chromatid exchanges, serum enzyme assays, and others), the most frequently used being comet assay [18,19,20,21,22,23]. Involvement of PARP in biomonitoring has been already reported [24,25,26].

In Campania, aregion in the South Italy, despite intervention programs for workers’ health defense include biomonitoring subjects at occupational risk of diseases, particularly cancer, little attention has been payed to monitor workers in agriculture. This field is so dynamic and complex (high number of agrochemicals, greenhouses or open cultures, different times of exposure, etc.) to make it difficult to follow it up.

From this area, we selected a group of volunteers (*n* = 82) in a healthy population of both people involved in agricultural work (in particular floriculturists, *n* = 42) and living/working close (*n* = 10) or far (*n* = 30, reference people) from greenhouses, to collect epidemiological data in order to determine prevalence of any disease, and study the possible correlation with pesticide-exposure by measuring PARP activity and automodification (PAR-PARP) in blood lymphocytes. Previous results allowed to establish that the test based on the determination of these parameters provides useful and early information for all those diseases that are associated with different levels of DNA damage [25]. The aim of this work is to verify the possible influence of the use of pesticides on human health, taking into account the hyperactivation and auto-modification of PARP as biomarkers of DNA damage. We propose this test in alternative to other molecular analyses [18,19,20,21,22,23], as possible, non-invasive, and routinar tools to monitor the healthy conditions of floriculturists.

## 2. Materials and Methods

### 2.1. Recruitment of Volunteers

Among floriculturist farms selected randomly on the basis of the register of local farmer associations, 42 volunteers directly exposed to pesticides (E), were recruited; 10 subjects of other working categories, including housekeeping women, indirectly exposed (IE), were asked to participate. The last group included 30 people enrolled at the Unit of Immunohematology and Transfusion of the Local Health Unit (ASL NA3, Torre del Greco, a town near Naples, Italy), and selected among blood donors (C, not exposed, negative controls). To increase the response rate, all volunteers, except those from the trans fusional center, were visited at home. 

The main groups, defined as directly exposed (E, floricoltorists), indirectly exposed (IE, no growers, living/working close to greenhouses), controls (C, not exposed, blood donors), underwent clinical and molecular analyses. All analyses were performed blindly and only at the end of the experiments the results were compared with anamnestic data. From this comparison, it was possible to define, within the E Group, three sub-groups of volunteers (Section 3.1 and Section 3.2). The three subgroups were indicated G1E, G2E, G3E, respectively on the basis of the criteria described in Section 3.3.

This work was within the research project approved by the BioeticCommettee “Carlo Romano”, Department of Public Medicine and Social Security, University “Federico II” of Naples (protocol no.131/11).

### 2.2. Questionnaire

All volunteers authorized to treat personal data anonymously and answered a questionnaire to collect personal data (name, age, gender), life style (diet, smoke, alcohol) and anamnesis (pharmacological treatments, recent infections, individual and familiar pathologies, etc.). Other items concerned occupational details. Subjects were asked to specify the exposure time inside greenhouses or storage areas per day and per month, the mixtures of employed agrochemicals, the way and frequency of pesticide spraying, and personal protective devices (gloves, boots, mask, etc.).

In all farms, the same pesticide mixtures were used.

Similarly, at recruitment, candidates to become blood donors had to fill a questionnaire with their general data (gender, age, job, life habits, etc.), and familiar/personal anamnesis, updated periodically. Then they underwent personal medical visits and diagnostic and clinical analyses to be admitted. We had access anonymous to general data. The transfusion center did not provide each personal questionnaire to guarantee donors’ privacy, but gave general information that all people we examined, met the requirements for admission and were negative for infectiousand serious cardiovascular diseases, diabetes, and serious allergy.

### 2.3. Clinical Analyses and Specimen Collection

Volunteers were subjected to conventional diagnostic analyses (determination of hematic glucose, nitrogen, cholesterol, triglycerides, transaminase activities, blood cell count) to assess health status at the time of enrollment. Blood samples of volunteers were obtained by venipuncture during home visit. Routinely, blood specimens were transferred to the Chemical Clinical Laboratory (ASL NA3) within two hoursfrom collection. The clinical analyses of blood donors were carried on at the Unit of Immunohematology and Transfusion (ASL NA3).

### 2.4. Lymphocyte Isolation and Lysis

Total lymphocytes in the blood sample were counted by (FACS, Bio-Rad, Milan, Italy). Lymphocytes were prepared according to protocol, provided with Ficoll (GE Healthcare, Milan, Italy). Two aliquots (1 mL) were used per each sample. Briefly each blood aliquot (1 mL) was layered on a Ficoll–Hypaque cushion (1:0.7, *v*/*v*) and centrifuged at 250× *g* for 10 min. Because of their lower density, the lymphocytes were found at the interface between the plasma and the Ficoll–Paque PLUS with other slowly sedimenting particles (platelets and monocytes). The lymphocytes were recovered from the interface and subjected to short washing steps to remove any platelets, Ficoll–Paque PLUS and plasma. Crude lymphocyte fraction was washed twice with 0.9% NaCl, followed by 10 mincentrifugation at 250× *g*. Pelleted pure lymphocytes were suspended in 0.9% NaCl (100 µL/blood mL). Few microliters of the suspension were used for counting recovered pure lymphocytes by FACS. In general, a 20% loss was measured. Pure cells were often used as freshly prepared fraction orstored at −80 °C until used. Cells from 1 mL blood were lyzed by suspension in lysis buffer (300 μL; 10 mM Tris-HCl pH 7.5, 1% Nonidet P40, 2 mM Spermidine-HCl, 10 mM Na_2_EDTA, protease inhibitor cocktail 2 μg/mL (Sigma-Aldrich, Milan, Italy), 1 mM Phenyl Methyl Sulphonyl Fluoride, PMSF) and incubation for 30 min at 4 °C. The whole lysate was further analyzed. Protein content was determined by Bradford’s reagent (Bio Rad, Milan, Italy) according to the provided instructions.

### 2.5. PARP Assay

PARP activity was assayed in duplicate in two cell preparations [27]. The reaction mixture (final volume 50 µL)contained 0.5 M Tris-HCl pH 8.0, 50 mM MgCl_2_, 10 mM DTT, 0.4 mM [^32^P]NAD^+^ (10,000 cpm/nmole) and a defined amount (20 µg protein)of whole lysates. After incubation for 15 min at 25 °C, the reaction was stopped by transfer onto ice and addition of 20% (*w*/*v*) trichloroacetic acid (final concentration). The mixture was filtered through Millipore filters (HAWPP0001, 0.45 µm) and washed with 7% trichloroacetic acid. The activity was measured as acid-insoluble radioactivity by liquid scintillation in a Beckman counter (model LS 1701, Milan, Italy). Values were expressed per 10^6^ cells.

### 2.6. SDS-PAGE, Immunochemical, and Densitometric Analysis

SDS-Polyacrylamide (12%) gel electrophoresis was according to Bianchi et al. [25]. Immunochemical analysis was performed blindly on blood samples collected from both healthy people and volunteers [28]. Polyclonal anti-PARP1 catalytic site antibodies (H-250, Santa Cruz Biotechnology, Inc., Dallas, TX, USA) were used to evidence PARPs, in either native or automodified state.

A control experiment was done to determine directly that the endogenous protein acceptor of poly(ADP-ribose) was PARP. Alkali incubation of PAR-PARP was carried on in 10 mM Tris-HCl pH 9.5, for three hours at room temperature, optimal conditions for completely removing the polymer from the acceptor protein. The alkali mixture was analyzed by SDS-PAGE and immunoblotting to evidence native PARP (data not shown). Automodified PARP was quantified by densitometric analyses of immunobands with a Chemidoc apparatus (Bio Rad, Milan, Italy) and expressed as optical density (OD, i.e., intensity of a band)/mm^2^.

To measure the densitometric value a rectangle was drawn encircling the band to quantify. The area of the rectangle was always constant. Therefore, the variable parameter was the intensity of the band. Even if the Quantity One program software automatically subtracted a mean value of the densities measured all over the filter (blank), a background value was manually subtracted. This manual blank was measured by using the above described rectangle on an area of the filter without bands. In addition, the results were normalized by performing densitometric measures of images with the same time of development and chemiluminescent capture signals, as determined by preliminary analyses. In this way, the results could be easily compared for all experiments.

### 2.7. Statistical Analyses

Chi-square test, ANOVA, and Dunnett’s post-hoc tests, non-parametric Mann–Whitney analysis were performed to determine the statistical significance of the results from the various groupsand they were compared by box-plot analysis [29,30].

## 3. Results and Discussion

### 3.1. Volunteers

Eighty-two people agreed to participate in the study. Forty-two were floriculturists frequenting greenhouses (E, exposed people), 10 were enrolled among subjects living close to or occasionally frequenting greenhouses (flower manipulators, transporters, housekeepings, students, etc.; IE, indirectly exposed), Table 1; 30 blood donors, involved neither directly nor indirectly with flower growth (never exposed, C, negative controls), were provided by the Trans fusional Center of Local Health Unit. They were selected from other work categories, not involved in culturing, and living far from cultured fields.

All volunteers, including controls, were Caucasic and recruited within the same district to normalize some parameters (food sources, climatic factors, environmental conditions, etc.). Most interviewed people had mild life habits (mixed diet, limited smoke, low/medium alcohol consumption), Table 1. The statistical meaning of the different number of males versus females in the various groups was evaluated by the chi-square test [30]. For *p* ≤ 0.05 the difference between male/female numbers among groups was not significant: the chi-square distribution calculated was below the critical value (*X^2^ = 4.06 < X^2^_critical value_ = 5.99*).

Considering that smoke can influence individual health, and that in the volunteer group there was a high percent of smokers, chi-square test was performedalso for smokers. For *p* ≤ 0.05 *X^2^* distribution of smokers between groups highly significant. Controls had a smaller number of smoking people and were included in the control group in order to consider individual variability, for smoke, frequent among volunteers.

Floriculturists (*n* = 42) belonged to families working in the field from at least threegenerations and/or were daily exposed to pesticides (spraying and presence in greenhouses 6–12 h a day). All of them used variable mixtures of the same pesticides (imidacloprid, abamectine, metomil, oxamil, deltametrine, endosulfan, spinosyns) and sprayed on Saturday to re-enter the greenhouses on Monday. Twenty-three growers used gloves/mask daily and only 14 ones worefull protective devices during spraying (Table 1).

### 3.2. Epidemiological Data

Personal anamneses and clinical results of volunteers were first compared with familiar anamneses on the basis of declared personal and/or familiar pathologies (Table 2). Each volunteer was cited as many times as the number of declared diseases.

Anamnestic data were recorded back to grandparents, since interviewed floriculturists belonging to the same families declared that culturing activity started at least two generations ahead.

By comparing familiar anamneses of IE (*n* = 10) and E (*n* = 42) groups, 10% and 28% families respectively were healthy. Comparable was the incidence of diabetes, HCV and cardiovascular diseases. Lung cancers occurred mainly in E group. No relevant incidence differences were highlighted analyzing personal anamneses.

### 3.3. PARP Activity

#### 3.3.1. PARP Activityin Controls

**Figure 1:**
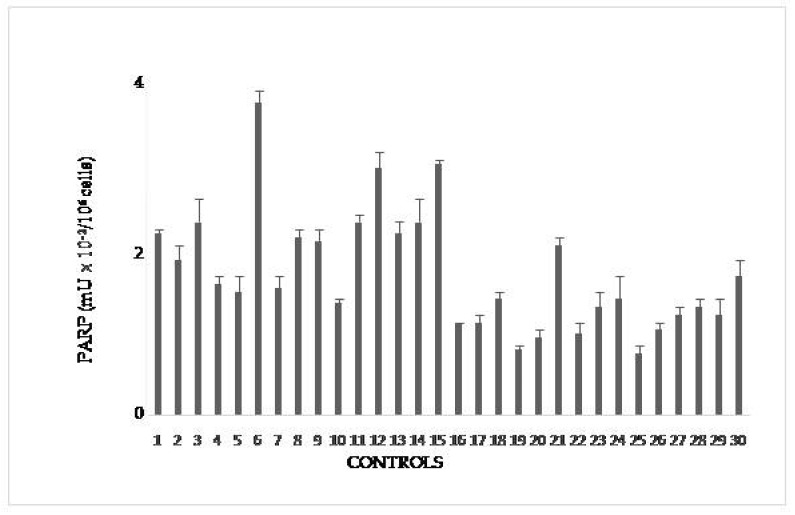
PARP activity in lymphocytes from blood donors. The values are mean averages of duplicate measures from two different lysate preparations (20 µg proteins per each assay).

In Figure 1 PARP activity of controls is reported in mU/10^6^ cells. Upper value is 4 mU/10^6^ cells and is taken as cut-off in the following figures. It is slightly higher than that in the range of physiological PARP reported in the literature(1.8–3.0 mU/10^6^ cells × 10^−2^). This difference allows to consider individual variability, possibly due to factors like alcohol consumption, temporary stress, and the above-evaluated smoke.

#### 3.3.2. PARP Activity in Volunteers

**Figure 2:**
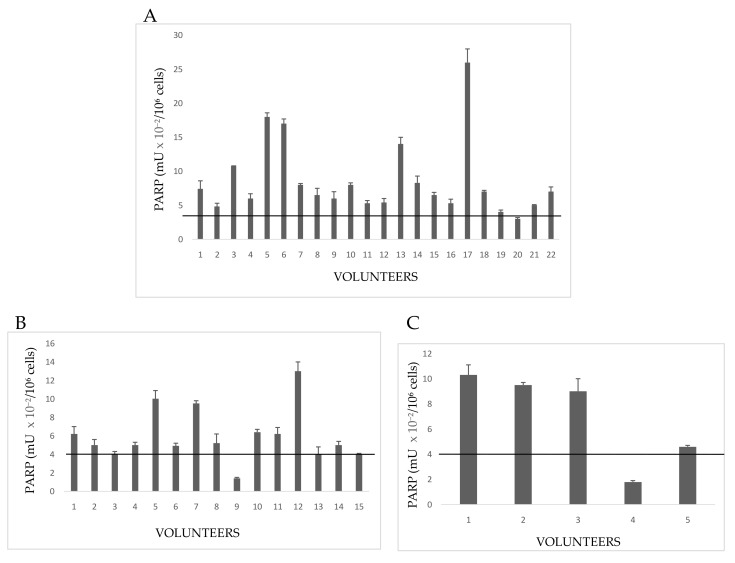
PARP activity in lymphocytes from Exposed People. (**A**) Volunteers with both familiar and personal pathologies; (**B**) subjects declaring only familiar diseases; (**C**) healthy people with neither familiar nor personal pathologies. The values are mean averages of duplicate measures from two different lysate preparations (20 µg proteins per each assay). The black line is the cut-off, the upper limit of PARP activity in controls (Figure 1).

PARP activity was evaluated by grouping floriculturists, daily exposed (E) to pesticides, on the basis of common anamnestic features (Figure 2). In particular, the feature considered was presence/absence of pathologies (Table 2).

Group 1E included 22 growers declaring both familiar and personal pathologies (Figure 2A). A number of these volunteers showed PARP activity levels above the cut-off of controls (no growers); in particular, hyperactivation of PARP in Subjects 3,5, 6, 13, and 17 spanned from three to six times the upper physiological limit determined for not exposed people (4 mU × 10^−2^/10^6^ cells). Other subjects, despite the declared diseases, showed a normal PARP activity. A possible explanation could be that once diagnosed the disease, these growers chose to monitor their health status by periodic checks and undergoing to appropriate therapies, which limited the dangerous effects of pesticides.

In Group 2E, all 15people had a familiar anamnesis with pathologies, but they themselves were healthy (Figure 2B). However, most floriculturists had altered PARP activity, increased to more than four times the control cut-off. By considering that those people were healthy on the basis of clinical and diagnostic reports, a high alteration of PARP might be interpreted as a response to a significant damage to DNA, possibly correlated with ex position to pesticides.

**Figure 3:**
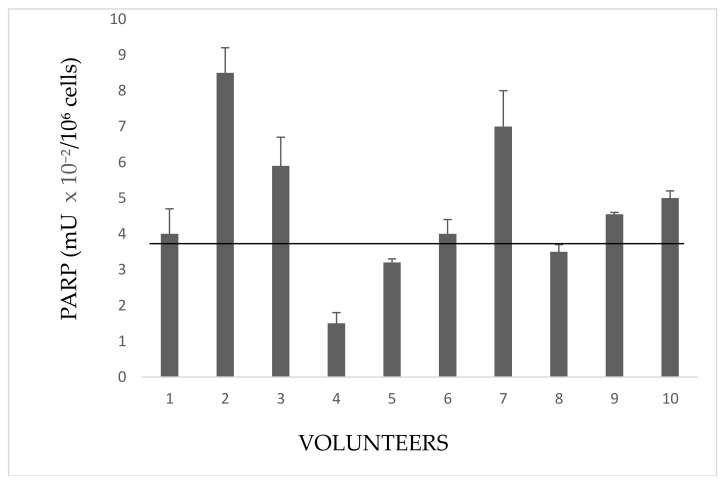
Activity in lymphocytes from indirectly exposed people. The values are mean averages of duplicate measures from two different lysate preparations (20 µg Proteins per each assay). The black line is the cut-off, the upper limit of PARP activity in controls (Figure 1).

Five volunteers (Group 3E) had neither familiar nor personal pathologies, but three of five subjects showed serious increase of PARP activity (Figure 2C). This result allows to hypothesize that, in the absence of diseases, possible causes of enzyme hyperactivation and, consequently, of DNA damage, might be both the prolonged (up to 12 h a day), and long-lasting (10–40 years) exposure to pesticides in greenhouses. PARP activity of indirectly exposed volunteers is shown in Figure 3.

These people frequent rooms near the greenhouses or live close to them. Subject 2 is a girl student, daughter of the farm owner, whose bed room overlooks the greenhouse. Subjects 3,6, and 7 handle and package flowers (they do not wear gloves). Subjects 1,5, and 8 are housekeepers living next to the greenhouse. Subjects 4 and 9 are parking attendant and secretary respectively, near greenhouses. Subject 10 occasionally helped in packaging flowers.

### 3.4. Immunoblottings and PAR-PARP Measure

#### 3.4.1. Immunoblottings

**Figure 4:**
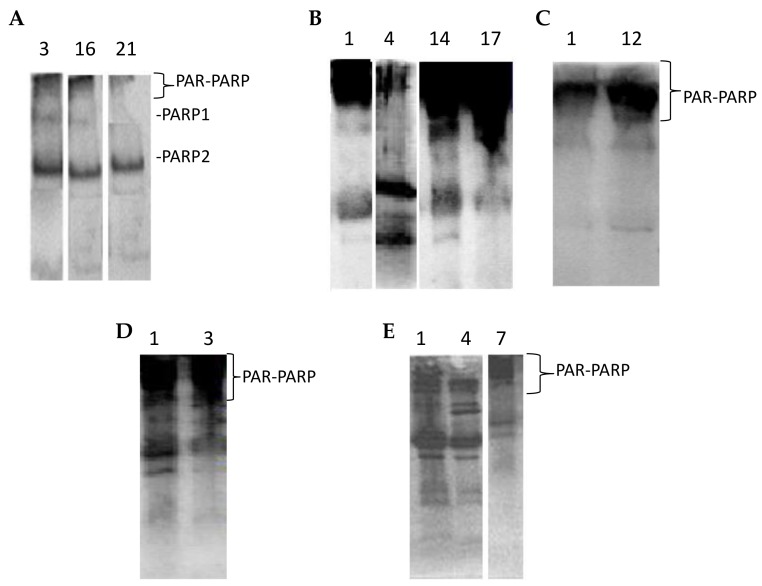
Anti-PARP immunoblottings of lysate cell proteins. (**A**) Controls; volunteers of (**B**) Group 1E; (**C**) Group 2E; (**D**) Group 3E; (**E**) Group IE. Numbers indicate volunteers as in Figure 1, Figure 2 and Figure 3. The same amount of proteins (20 µg) was loaded for all samples.

Anti-PARP immunoblottings of all analyzed samples were performed to confirm PARP activity results and to evidence and measure the levels of endogenous PARP automodification (PAR-PARP). Figure 4 shows some lymphocyte immunopatterns of controls, exposed and indirectly exposed people. The densitometric analysis of the region corresponding to automodified PARPs, started just above 113 kDa up to the top of the filter, slightly above the mobility of the first molecular weight marker (205 kDa). It was not possible to differentiate the modification levels of both PARP 1 and PARP 2; we could only look at the amount of native PARPs still present.

In lymphocytes, under physiological conditions, PARP 2 is more abundant compared to PARP 1 (Figure 4A). Only traces of PAR-PARP are detectable in controls.

Automodified PARP increases in immunoblots from both exposed and indirectly exposed people (Figure 4B–E).

#### 3.4.2. PAR-PARP Measurement

**Figure 5:**
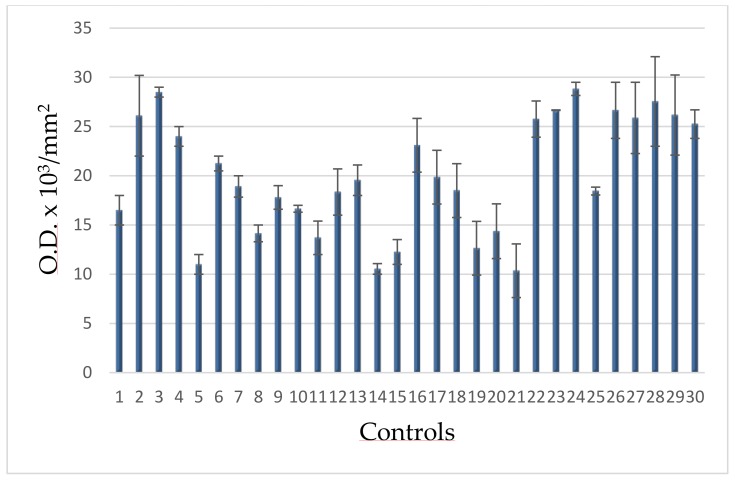
PAR-PARP levels of blood donors.

The results of densitometric analyses performed for all volunteers were grouped as for PARP activity (Section 3.3). In Figure 5 densitometries of controls are reported.

The range of these values allowed to calculate the upper limit (30 O.D. × 10^3^/mm^2^), taken as cut-offand reported in the following figures.

**Figure 6:**
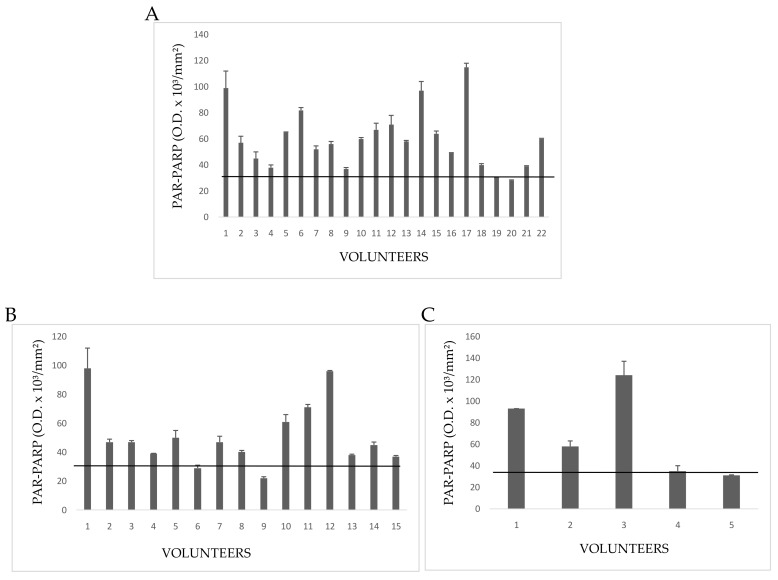
PAR-PARP values of exposed subjects. The black line is the cut-off. Mean values of duplicate measures from two different lysate preparations. (**A**) G1E group; (**B**) G2E group; (**C**) G3E group.

The values for the three groups of directly exposed people showed the same trend of those of PARP activity (Figure 6).

In Group 1E five people had PAR-PARP values slightly above the cut-off (Figure 6A). The other subjects showed PAR-PARP levels corresponding to DNA damage from moderate to excessive; in Volunteers 5, 12, 15, 19, and 22 PARP hyper-modification reached the maximal levels indicating an extensive DNA damage. Such high levels could not be explained on the basis of declared personal pathologies, since other people affected by the same diseases had moderate or even nearly physiological PAR-PARP. As a hypothesis, PARP hyper-modification for Dubjects 5, 12, 15, 19, and 22 might correlate to the years (10–40) of greenhouse frequency.

Group 2E included 15 growers with many years of activity in greenhouses, and declaring no personal diseases, even if they have pathological familiar anamnesis (Figure 6B). Relevant PAR-PARP results were found in Subjects 2, 3, 8. In the absence of pathologies, such high levels might be explained better with daily and long periods of greenhouse frequentation.

The five subjects of Group 3E declared to be healthy for both familiar and personal anamneses (Figure 6C). However, two of them had PAR-PARP above 60 O.D/mm^2^. As for Group 2E it can be hypothesized that this PARP hypermodification might be due to an uninterrupted and prolonged exposition to pesticides.

**Figure 7:**
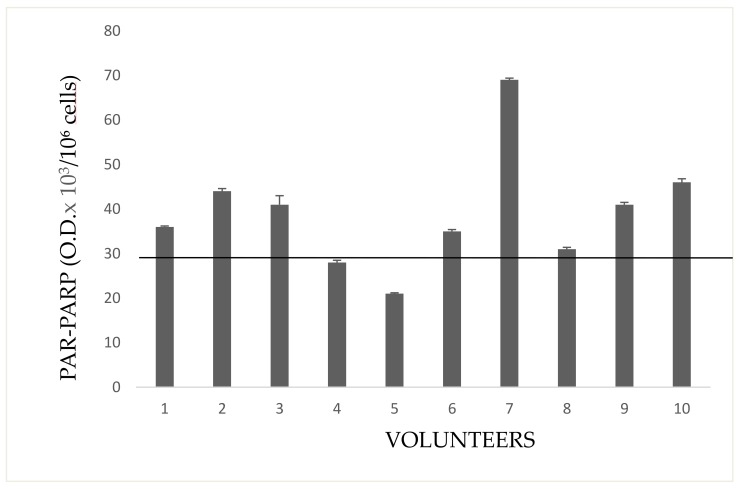
PAR-PARP in indirectly exposed subjects. The black line is the cut-off. Mean values of duplicate measures from two different lysate preparations.

Indirectly exposed volunteers, frequenting rooms next to greenhouses (storekeepers, secretary, staff for the packaging of flowers), or living close to them, are shown in Figure 7. In general, most growers, have houses in proximity to greenhouses. Only the subject no. 2 showed a very high PAR-PARP. This subject had bed room overlooking a greenhouse.

### 3.5. Statistical Analyses

The data of PARP activity and PAR-PARP were checked with a double statistical analysis, the parametric ANOVA test and the non-parametric Mann–Whitney U test.

Anova test gave, for both parameters, a calculated variance ratio largely above the expected one (for α = 0.05), confirming the statistical significance of results from all the different groups. By using the ANOVA data, the Dunnet’s post-hoc test, was carried out (α < 0.05). Among different post-hoc tests, this one allows to better compare control with other groups, and to check whether there is any group not showing a significant difference with control. Once calculated the critical distance between means with data (PARP activity and PAR-PARP) of control group, the distance between the means of each volunteer’s group and that of controls was always higher. Therefore, the distances of all groups were statistically significant.

The non-parametric Mann–Whitney U test was also performed because of the small sizes of the groups (≤30) and the presence of outliers in some samples. This test allowed to evaluate the difference of the groups on the basis of medians instead of means. Pairs of groups were compared, in order to determine the U value (*p* ≤ 0.05). In each pair controls were compared with one of the volunteers’ groups. The calculated U values were all above the corresponding tabulated critical one, and again confirmed the statistical significance of results in the various groups.

**Figure 8:**
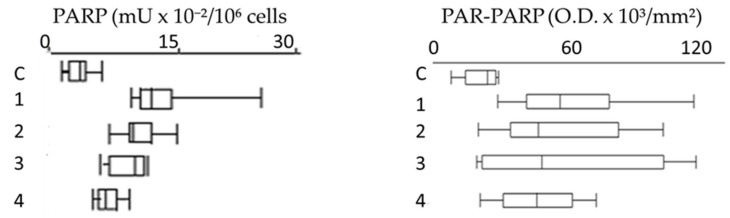
Box plots of PARP activity and PAR-PARP levels in different groups. C, Controls; 1, Group 1E; 2, Group 2E; 3, Group 3E; 4, indirectly exposed.

In order to evaluate better the collected data, the results of PARP activity and PAR-PARP measure were compared by the box plot analysis (Figure 8).

Compared to controls, all groups show that PARP hyperactivation and PAR-PARP levels follow the same trend, indicating from moderate to high DNA damage. In Group 1E the occurrence of both familiar and personal pathologies, and possible therapies, does not allow to explain directly the damage withthe use of pesticides (Figure 8).

Most significant are the results of plots 2 and 3 (Group 2E and 3E, respectively)thatsuggest anextensive alteration of PARP activity and PAR-PARP levels and thereby a massive DNA damage. In the absence of personal pathologies, it is conceivable that such high values might be explained with activity in greenhouses and prolonged exposition to pesticides. These are the most fitting examples that monitoring of growers with these biomarkers can be determinant in highlighting the alteredconditions that clinical and diagnostic analyses are not able to demonstrateso early.

## 4. Conclusions

This research highlights the importance of PARP as biomarker of DNA damage, a simpler alternative to other methods to measure DNA damage [13,14,15,16]. As it regards PARP activity and PAR-PARP levels, our study confirmed their general meaning as DNA damage markers. Even before performing other more complex diagnostic analyses, and in accordance with data from literature, DNA damage is easily detectable by these measures.

We like to underline that the presented results do not allow to draw conclusive remarks as they need a larger sampling to get statistically significant data for both other workers and pesticide exposed people. However, they suggest a trend: where there are no known pathologies, there are signs, namely PARP biomarkers (PARP hyperactivation and PAR-PARP), that may suggest more frequent controls of growers and possible therapeutic interventions. Thereby, we propose PARP activity and auto-modification in peripheral blood lymphocytes as possible, non-invasive, and routinar tools to monitor the healthy conditions of floriculturists.

In perspective, once validated, these simple and noninvasive molecular methods could be employed in planning a constant biomonitoring of workers, not only floriculturists, occupationally exposed to risk.

## Figures and Tables

**Table 1:** General features of enrolled population and controls.

	Exposed ^a^	Indirectly Exposed ^b^	Not Exposed (Controls)
Number (*n*)	42	10	30
Age Median (IQR)	43 (19)	34 (17)	42 (14)
Males	17	7	20
Females	25	3	10
Smokers	26	4	8
Years of exposure, median (IQR)	15 (12)	-	-
Pesticide exposure (hours/day)	2–12	-	-
Preparation and spraying of pesticide mixtures	29	-	-
Full protective devices (only for spraying)	14	-	-
Daily use of gloves and/or mask	23	-	-
No protection	16	-	-

^a^ All working in greenhouses with ornamental crops. ^b^ Living/working daily (handling and packaging flowers) close to green houses.

**Table 2:** Pathologies declared and diagnosed in exposed and indirectly exposed people.

	Familiar Anamnesis		Personal Anamnesis	
	IE (*n* = 10) E (*n* = 42)		IE (*n* = 10) E (*n* = 42)	
	Frequency ^a^		Frequency ^a^	
*Pathology*				
None	1	12	5	20
Lung disease ^b^	0	2	1	3
Neuropathy ^c^	1	12	1	0
Diabetes	3	13	0	3
Thyroidism	0	3	2	6
Cardiovasc. d. ^d^	5	15	0	4
HCV ^e^	1	5	0	1
Cirrosis	0	2	0	1
Gut d./*H.pylorii*	0	0	0	1
*Malignant disease*				
Uterus	0	1	0	1
Prostate	0	0	2	1
Lung	1	6	0	0
Pancreas	0	1	0	1
Colon/gastric	0	1	0	0
Bladder	0	1	0	0
Liver	0	2	0	2
Leukemia	0	1	0	0
Brain	0	0	0	1

^a^ Times of occurrence. Each volunteer was cited as many times as the number of declared diseases. ^b^ Asthma, allergy. ^c^ Stroke, ischemia, depression. ^d^ Hypertension, angina, heart attack. ^e^ Hepatitis C Virus.
